# Supplementary material for: Long-Term Oocyte-Like Cell Development in Cultures Derived from Neonatal Marmoset Monkey Ovary
Source: Stem Cells Int. 2015 Nov 9;2016:2480298. doi: 10.1155/2016/2480298 (PMC4655298; doi:10.1155/2016/2480298)
Supplement: Supplementary file 1 — Supplementary Figure 1. The number of reads per sample analyzed in this study by transcriptome analysis. ES cells (samples 1-4), fibroblasts (samples 5-10), ovarian cell culture samples (samples 11 and 12), and native marmoset monkey neonatal ovaries (samples 13 and 14) were analyzed. The numbers of reads per sample were between 13 and 19 × 106. Supplementary Figure 2. The number of detected transcripts per sample analyzed in this study by transcriptome analysis. Around 40.000 transcripts were detected in all samples [ES cells (samples 1-4), fibroblasts (samples 5-10), ovarian cell culture samples (samples 11 and 12), and native neonatal ovaries (samples 13 and 14)]. Supplementary Figure 3. The cultured ovarian cells were highly proliferative at low passages (P1, left panel) as revealed by Ki-67 staining. At higher passages (P9), OCCs showed a reduced number of Ki-67-positive cells. Highly proliferative ES cells were used as positive control. The primary antibody (#9027S from Cell signaling Technology) was used in a 1:300 dilution. The scale bar represents 50 μm. Supplementary Table 1. Ovary vs. OCCs top 50 up-regulated genes corresponding to data base identifier. [file 2480298.f1.zip › 2480298.f1/Fereydouni_et_al__Stem_Cells_International_Resubm_final_SCI_1421212.pdf]

Long-term oocyte-like cell development in cultures derived from neonatal marmoset monkey ovary

Bentolhoda Fereydouni[1], Gabriela Salinas-Riester[2], Michael Heistermann[3], Ralf Dressel[4], Lucia Lewerich[1], Charis Drummer[1], Rüdiger Behr[1]

[1]Stem Cell Biology Unit, German Primate Center – Leibniz Institute for Primate Research, Kellnerweg 4, 37077 Göttingen, Germany
[2]Microarray and Deep-Sequencing Core Facility, University Medical Center Göttingen (UMG), Justus-von-Liebig-Weg 11, 37077 Göttingen
[3]Endocrinology Laboratory, German Primate Center – Leibniz Institute for Primate Research, Kellnerweg 4, 37077 Göttingen, Germany
[4]Department of Cellular and Molecular Immunology, University of Göttingen, Humboldtallee 34, 37073 Göttingen, Germany

Author contribution:

Bentolhoda Fereydouni:
Conception and design, Collection and assembly of data, Data analysis and interpretation, Manuscript writing, Final approval of manuscript

Gabriela Salinas-Riester:
Collection and assembly of data, Data analysis and interpretation, Manuscript writing, Final approval of manuscript

Michael Heistermann:
Collection and assembly of data, Data analysis and interpretation, Final approval of manuscript

Ralf Dressel:
Financial support, Collection and/or assembly of data, Final approval of manuscript

Lucia Lewerich:
Collection and/or assembly of data, Data analysis and interpretation, Final approval of manuscript

Charis Drummer:

Provision of study material, Final approval of manuscript

Rüdiger Behr:

Conception and design, Financial support, Collection and/or assembly of data, Data analysis and interpretation, Manuscript writing, Final approval of manuscript

Corresponding author's address:
Rüdiger Behr
German Primate Center
Stem Cell Biology Unit
Kellnerweg 4
D-37077 Göttingen
Germany
Fon: +49 551-3851-132
Fax: +49 551-3851- 431
Email: rbehr@dpz.eu

Declaration of interest

The authors declare that there is no conflict of interest that could be perceived as prejudicing the impartiality of the research reported.

Funding

Bentolhoda Fereydouni is on a scholarship of the Deutscher Akademischer Austauschdienst (DAAD). The work was mainly supported by institutional resources and marginally by a grant of the Deutsche Forschungsgemeinschaft (DFG; Be2296-6) entitled "Pluripotent cells from marmoset testis" to Rüdiger Behr. The cell injection assays in mice performed by Ralf Dressel were supported by a Collaborative Research Centre from the Deutsche Forschungsgemeinschaft (SFB1002, TP C05).

Key words: ovary, oogonia, germline stem cell, VASA, non-human primate

Abstract

We use the common marmoset monkey (*Callithrix jacchus*) as a preclinical non-human primate model to study reproductive and stem cell biology. The neonatal marmoset monkey ovary contains numerous primitive premeiotic germ cells (oogonia) expressing pluripotent stem cell markers including OCT4A (POU5F1). This is a peculiarity compared to neonatal human and rodent ovaries. Here, we aimed at culturing marmoset oogonia from neonatal ovaries. We established a culture system being stable for more than 20 passages and 5 months. Importantly, comparative transcriptome analysis of the cultured cells with neonatal ovary, embryonic stem cells and fibroblasts revealed a lack of germ cell and pluripotency genes indicating the complete loss of oogonia upon initiation of the culture. From passage 4 onwards, however, the cultured cells produced large spherical, free-floating cells resembling oocyte-like cells (OLCs). OLCs strongly expressed several germ cell genes and may derive from the ovarian surface epithelium. In summary, our novel primate ovarian cell culture initially lacked detectable germ cells, but then produced OLCs over a long period of time. This culture system may allow a deeper analysis of early phases of female primate germ cell development and - after significant refinement – possibly also the production of monkey oocytes.

Introduction

It is a long-held opinion in reproductive biology that in females of most species, including the human, the postnatal germ cell pool is limited in number and cannot be replenished or even expanded. Early studies in the rat showed that oogonia, the proliferating female germ line progenitor cells that enter meiosis to produce oocytes, were found only during fetal development [1]. Human females are also thought to be born with a non-renewable pool of germ cell follicles which declines with age [2]. In postnatal human ovaries, oogonia were found only very sporadically and were undetectable by the age of two years [3]. A lack of evidence for postnatal germ cell proliferation in human ovaries was also reported by Liu, *et al.* [4]. Instead, by far most of the proliferation-competent oogonia in the human ovary entered meiosis by the end of the second trimester of gestation [5, 6]. Recent studies using transgenic mice and non-human primates also reported a lack of evidence for germ cell proliferation in adult mouse [7, 8] and monkey [9] ovaries. However, these data supporting the view of a fixed postnatal female germ cell pool are profoundly challenged by reports starting a decade ago [10, 11] , suggesting replenishment of the ovarian germ cell pool in mice. The existence of mitotically active germ line stem cells in postnatal mouse and human ovaries was suggested by the isolation, molecular characterization, and transplantation of cell populations [10, 12]. Probably the strongest evidence so far for a germ line stem cell pool in the ovary was provided by Zou, *et al.* [13], who generated mouse female germ line stem cells (FGSCs) with the ability to reconstitute oogenesis and produce offspring after transplantation. Other authors reported the production of oocyte-like cells *in vitro* from cultures of adult ovarian surface epithelium (OSE) [14-16]. In summary, currently there are several apparently contradictory reports which provide data supporting or negating generation of new oocytes in the postnatal rodent and primate - including the human - ovary. Adding to this complex situation, Dyce, *et al.* [17] reported that even fetal porcine skin cells can form oocyte-like cells (OLCs) *in vitro*.

We use the common marmoset monkey (*Callithrix jacchus*) as a non-human primate model to study reproductive and stem cell biology. In contrast to other species used in reproductive biology, the marmoset monkey still has numerous oogonia in the neonatal ovary, which robustly express the pluripotency associated markers OCT4A, LIN28 and SALL4, the germ cell marker VASA and the proliferation marker KI-67 [18]. Therefore, these oogonia in the neonatal marmoset ovary share important pluripotency markers with marmoset monkey embryonic stem (ES) cells [19]. We failed to detect this proliferating and pluripotency-marker-positive cell population in the one-year-old and adult ovary [18] indicating a fast postnatal oogonial clearing also in this non-human primate species. Here, we report studies on the culture of neonatal marmoset ovarian cells. We originally aimed at culturing the proliferating marmoset monkey oogonia exhibiting a pluripotency signature similar to marmoset monkey embryonic stem cells. Hence, we established a mouse embryonic fibroblast (MEF)-based cell culture system, which allowed the long-term culture of ovarian cells. However, germ cell and pluripotency marker expression was lost in the first passages indicating the complete loss of the oogonia. After a few passages, however, germ cell marker expression recovered, and we observed in later passages the development of large spherical, free-floating cells which we called oocyte-like cells (OLCs) due to their morphological resemblance with the cells reported by Dyce, *et al.* [17]. OLCs had a diameter up to ~40 µm and strongly expressed several germ cell markers. This study demonstrates the development of oocyte-like cells in long-term ovarian cell cultures from a translational and experimentally accessible non-human primate species.

Materials and methods

Animals

Marmoset monkeys (*Callithrix jacchus*) for this study were obtained from the self-sustaining breeding colony of the German Primate Center (Deutsches Primatenzentrum; DPZ). The German Primate Center is registered and authorised by the local and regional veterinary governmental authorities (Reference number: 122910.3311900, PK Landkreis Göttingen). Health and well-being of the animals were controlled daily by experienced veterinarians and animal care attendants. The legal guidelines for the use of animals and the institutional guidelines of the DPZ for the care and use of marmoset monkeys were strictly followed. The animals were pair-housed in a temperature- (25 ± 1 °C) and humidity-controlled (65 ± 5%) facility. Illumination was provided by daylight and additional artificial lighting on a 12.00:12.00 hour light: dark cycle. The animals were fed ad libitum with a pelleted marmoset diet (ssniff Spezialdiäten, Soest, Germany). In addition, 20 g mash per animal was served in the morning and 30 g cut fruits or vegetables mixed with noodles or rice were supplied in the afternoon. Furthermore, once per week mealworms or locusts were served in order to provide adequate nutrition. Drinking water was always available.

In captivity, marmosets sometimes give birth to triplets or even quadruplets. However, the mother is usually able to feed and rear only two neonates, which is the normal litter size of free-living marmosets. Therefore, the neonates from triplet births were used to collect organs for this study. Marmoset monkey ovaries were obtained from six neonatal animals (postnatal days 1-5). All animals were narcotized with Pentobarbital (Narcoren®; 0.05 ml intramuscular) and euthanized by an experienced veterinarian with an intracardial injection of 0.5 ml Pentobarbital before a lack of nourishment caused suffering of the animals. Wherever applicable, the ARRIVE guidelines were followed.

Numbers of animals

Altogether, ovaries from 6 neonatal marmosets were used in this study: 5 pairs for culture and one pair as reference for transcriptome analysis.

Neonatal common marmoset monkey ovarian cell cultures

Neonatal ovaries (approximate dimensions 2 mm x 1 mm x 1 mm, see also [18]) were first collected and washed in a petri dish containing cold DPBS. Fat and blood cells were removed and the whole ovary including the OSE was transferred to fresh buffer and minced with sterile scissors. Minced ovaries were then transferred to a 15 ml falcon tube. After gentle centrifugation, DPBS was removed and ovarian tissue fragments were re-suspended in DMEM/F12 medium containing collagenase (Sigma#C2674) and DNase and kept for 45 minutes at 37°C. Every 5 to 10 min the ovaries were gently pipetted to disintegrate the tissue. Finally, 10% FBS was added to inactivate the enzyme. In order to remove larger undigested tissue fragments, the cells were passed through 70µm strainer (BD, USA) and were centrifuged at 200g for 10 min. Then the supernatant was removed and the cells were re-suspended in culture medium and transferred on a prepared feeder layer of mouse embryonic fibroblast (MEF) feeder at a concentration 3-5 $\times 10^5$ cells per 5cm well. After one week large colonies were individually picked with a micro probe (FST, Heidelberg, Germany, # 10032-13) and digested with Accutase (Life Technologies, Germany) for 4 min. Then the cells were centrifuged at 300g for 10 minutes and re-suspended in the culture medium for further passages. Cultures were maintained at 37°C under 5% CO_2 and 5% oxygen in a humidified incubator.

Culturing neonatal common marmoset ovaries

Different cell culture conditions were tested. Finally this condition was selected: DMEM/F12 supplemented with FBS (10%), Pen/Strep, amphotericin B, and human LIF (10µg/ml) on an irradiated mouse embryonic fibroblast (MEF) feeder cell layer. For further passages (usually

after 7-10 days) colonies were mechanically removed with a sterile probe, digested with Accutase and placed on a newly prepared MEF layer.

Culturing marmoset monkey embryonic stem cells

Marmoset monkey ES cells were basically cultured as described previously [20]. Only for passaging of the ES cells, StemPro Accutase (Life Technologies) was used instead of trypsin–EDTA with subsequent mechanical dissociation.

Immunofluorescence staining

For immunofluorescence staining, medium was removed and cell colonies were washed twice in PBS and then fixed in 4% PFA for 15 minutes. Cells were permeabilized by 0.1% Triton X-100 in PBS for 10 minutes at room temperature. Primary antibody (VASA, R&D Systems, AF2030) was diluted in 3% BSA-PBS, and colonies were incubated for 1 h at 37 °C. Cells were washed twice in PBS. The secondary antibody, which was diluted in 3% BSA-PBS, was added to the cells and incubated for 20 minutes in a dark box at 37 °C. Cells were washed again twice in PBS and 5% DAPI-PBS was added. Cells were washed again with PBS and mounted with Citiflur (Science services AF1 Glycerol / PBS solution). For negative controls, the primary antibody was (i) omitted or (ii) replaced by IgG isotopes. DAPI staining of the OLCs was performed in 5% DAPI-PBS for 5 minutes followed by two washes in PBS.

Histology and immunohistochemistry staining

Histology of the colonies was performed after mechanically detaching of whole MEF layer including the ovarian cell colonies from the cell culture well. After detachment, the randomly arranged MEF layer was fixed in Bouin's solution for 3 hours, washed several times for at least 24 h in 70% EtOH and then embedded in paraffin wax. The resulting cell culture

conglomerate was randomly sectioned. Due to the large number of colonies sufficient sections of ovarian cell colonies in different orientations were available for histological analysis. Immunohistochemistry was performed as described recently [18] using the following primary antibodies: OCT4A (#2890S, Cell Signaling Technology, Germany; 1:100), LIN28A (#3978S, Cell Signaling Technology; 1:100–1:200), SALL4 (#ab57577, Abcam, UK; 1:200), VASA (DDX4; #AF2030, R&D Systems, Germany; 1:100).

Western blot analysis

Western blotting was performed according to standard procedures. In brief, 20 mg of frozen tissue per sample were used. Proteins were isolated by mechanical destruction of the tissue in a tissue lyser at 50 HZ (Qiagen, Hilden, Germany). The samples were denatured for 5 minutes at 95°C. Samples were then run on a 10% SDS-page gel in Tris HCl buffer, pH 8.8, and then semi-dry blotted onto a PVDF membrane (150 mA for 1h). Blocking of unspecific binding was achieved by incubating the membrane in 5% skim milk powder diluted in TBS for 1 h. Primary antibodies against VASA (#AF2030, R&D Systems; 1:2000) and β-ACTIN (Santa Cruz, SC-1616-R; 1:5000) were diluted in blocking buffer. Membranes were incubated in primary antibody solutions for 16 hours at 4°C and then washed three times with blocking solution supplemented with tween 20. After incubation with the horseradish peroxidase-coupled secondary antibody (1 h at 20°C) the membrane was washed again. Detection of bound antibody was performed using the Amersham ECL Western Blotting Detection Reagents kit (RPN2106). Signals were detected and documented using the ChemoCam Imager (INTAS, Göttingen, Germany).

Transcriptome Analysis

For transcriptome analysis, two neonatal marmoset ovaries, colonies from P4 from two different individual animals (100 - 300 colonies per sample), marmoset skin fibroblasts and

marmoset ES cells were analyzed. Primary fibroblasts were obtained and cultured as described recently [21]. RNA was isolated using the TRIzol® Reagent (Life Technologies) according to manufacturer's instructions. RNA quality was assessed by measuring the RIN (RNA Integrity Number) using an Agilent 2100 Bioanalyzer (Agilent Technologies, Palo Alto, CA). Library preparation for RNA-Seq was performed by using the TruSeq RNA Sample Preparation Kit (Illumina, Cat. N°RS-122-2002) starting from 500 ng of total RNA. Accurate quantitation of cDNA libraries was performed by using the QuantiFluor™ dsDNA System (Promega). The size range of final cDNA libraries was determined applying the DNA 1000 chip on the Bioanalyzer 2100 from Agilent (280 bp). cDNA libraries were amplified and sequenced by using the cBot and HiSeq2000 from Illumina (SR; 1x50 bp;5- 6 GB ca. 30-35 million reads per sample). Sequence images were transformed with Illumina software BaseCaller to bcl files, which were demultiplexed to fastq files with CASAVA v1.8.2. Quality check was done via fastqc (v. 0.10.0, Babraham Bioinformatics). The alignment was performed using Bowtie2 v2.1.0 to the cDNA for *Callithrix jacchus.* Data were converted and sorted by samtools 0.1.19 and reads per gene were counted via htseq version 0.5.4.p3. Data analysis was performed by using R/Bioconductor (3.0.2/2.12) loading DESeq, gplots and goseq packages. Candidate genes were filtered to a minimum of 4x fold change and FDR-corrected p-value <0.05. The data discussed in this paper were generated in compliance with the MIAME guidelines and have been deposited in NCBI's Gene Expression Omnibus and are accessible through GEO Series accession number XXXXX (will be completed upon acceptance of the MS).

Quantitative Reverse Transcriptase Polymerase Chain Reaction (RT-qPCR) analysis

RT-qPCR was carried out as described previously [18]. Total RNA was extracted from different passages of colony-forming cells. Around 50-100 colonies per passage were pooled and analyzed. Three independent cultures (derived from three different animals) were analyzed.

Each RNA sample was analyzed in triplicate. As positive controls, neonatal marmoset ovary and marmoset embryonic stem cells were used. Marmoset monkey fibroblasts served as a biological negative control for pluripotency and germ cell markers. Primers are listed in Supp. Table 1. For oocyte-like cell RNA extractions, altogether 28 cells from different passages were collected and randomly divided into two groups. The RNA was isolated with the RNeasy MICRO kit (Qiagen) according to manufacturer's instructions. To analyze the relative gene expression level changes during the culture within one passage, colonies from P7 were seeded into 8 separate wells. The cells from two wells were harvested for analysis at day 2, 4, 6, and 8.

Hormone measurements

Measurement of progesterone in the selected cell culture medium samples after at least 3 days of conditioning was performed using an enzyme immunoassay (EIA) using antiserum raised in sheep against progesterone-11-hemisuccinate-BSA as described by Heistermann and colleagues [22]. Fresh medium was used as control. Estradiol-17ß was determined using an EIA according to Heistermann et al. [23] with the exception that 17ß-estradiol-6-horse-radish-peroxidase was used as label. Both steroid measurements were performed in undiluted samples and fresh medium was used as control.

Cell transplantation assay

In order to test the cultured ovarian cells for their regenerative and tissue neomorphogenesis potential, we subcutaneously injected ~ 10^6 cells per mouse. The cells were obtained from Accutase-treated ovarian cell colonies from the 5th passage. Four female adult RAG2$^{-/-}\gamma c^{-/-}$ mice lacking B, T, and NK cells were used. The technical procedure has been described previously for neonatal testis tissue [24].

Results

General observations and morphology of the primary cell cultures

We established a long-term primary culture of ovarian cells. This included passaging as well as expansion of the cells. Five individual primary cultures of neonatal marmoset ovaries were performed and run up to 6 months with very similar morphology and kinetics. The maximum passage number was 23. Then the cells stopped proliferation. Initially, relatively small ovarian cell colonies (OCCs) formed which could be distinguished from the MEFs by the morphology of the cells and the colonies' boundaries (Fig.1A). The OCCs quickly increased in size forming big colonies with diameters up to 1000 µm (Fig.1B) within a few days. The cells forming the OCCs exhibited an epitheloid phenotype as judged from morphology (Fig.1D), i.e. they are apparently polar with apical nuclei, and no intracellular matrix was visible between the cells forming the colonies by light microscopy. However, the cells lacked typical epithelial markers such as E-Cadherin (CDH1). For further details, see below.In higher passages the individual OCCs became smaller (compare Fig.1B and C). Some OCCs developed in their centers a second layer of cells on top of the primary cell layer (Fig.1C). The morphology of the colonies was faintly reminiscent of primate ES cell colonies [20], (Fig.1E). Colonies with the morphology of OCCs were never observed in pure mouse embryonic feeder cell cultures.

OCCs lack a germ cell population in early passages

In order to initially compare the neonatal ovary-derived cell colonies on the transcriptome level with reference samples we performed a comprehensive transcriptome analysis of OCCs by deep sequencing and compared the data set with the transcriptomes of neonatal ovaries, which served as starting material and contained oogonia, marmoset ES cells as a reference for pluripotent cells, and skin fibroblasts, which represent prototypic mesenchymal cells. Each

individual sample's transcriptome was represented by at least 12 Mio. reads (Suppl. material Fig.1). About 40.000 different transcripts were detected in each individual sample (Suppl. material Fig.2). We used low passage number samples (passage 4) in order to obtain data from cells without extensive cell culture adaptation artefacts. Due to the very limited material, only two independent OCC samples and two neonatal ovaries could be analyzed. However, already this small set of samples provided valuable insights (Fig.2). The OCC samples were clearly distinct from native ovary and fibroblasts (Fig.2A). In contrast, the differences between the OCCs' and the ES cells' transcriptomes were smaller. Importantly, the PCA plot (Fig.2A) indicates fundamental differences between the transcriptomes of ovaries and OCCs. Notably, the top 50 differentially expressed genes between native ovaries and OCCs revealed two major facts (Fig.2B): 1. Almost all differentially expressed genes were overrepresented in the native ovary. This suggests that the OCCs generally represent a subpopulation of the whole cell population constituting the native ovary and that no completely different or novel cell type developed in culture, at least not in detectable quantities. 2. Among the top 50 differentially expressed genes are numerous germ cell-specific genes like *MAEL, RNF17, TEX12*, *TDRD9, MOV10L1, NOBOX, ZP3, FIGLA*, *SOHLH2*, *DAZL,* and *SYCP2*. In fact, several germ cell genes, including *DAZL*, *MAEL, RNF17, TEX12, TEX101,* and *TDRD* were totally undetectable in the transcriptomes of the OCCs. Other transcripts like *OCT4, LIN28,* and *VASA* were also extremely low or absent. This strongly indicates the complete loss of the typical neonatal ovarian germ cell population, including post-migratory PGCs, oogonia, and oocytes in the cell culture.

The comparison between the OCCs and the fibroblasts revealed an increased expression of many genes in the OCCs (Fig.2C). The up-regulated genes include *COL2A1*, the keratine gene *KRT36,* and *VCAM1*. Importantly, however, no germ cell gene was found up-regulated in OCCs compared to fibroblasts further substantiating the absence of germ cells from the

OCC cultures. The comparison of the top 50 differentially expressed genes between the OCCs and the ES cells showed that most genes were up-regulated in ES cells, like *TDGF1*, *LIN28A*, *OCT4* (*POU5F1*), and *NANOG*. Only a few genes including the dual specificity phosphatase 13 (*DUSP13*) and the serine peptidase inhibitor, Kazal type 1 (SPINK1) were up- regulated in OCCs (Fig.2D).

The detailed cellular identity of the OCCs could not be determined so far. Many of the cells of the OCCs were proliferation marker Ki-67-positive (Supplemental Fig. S3). Although the cells had an epitheloid shape, we failed to detect E-Cadherin in the transcriptome data as well as by IHC (data not shown). Also cytokeratins as characteristic proteins of epithelial cells were only barely represented in the transcriptomes. Vimentin as typical protein of cells of mesenchymal origin was expressed at medium levels (~50% of neonatal ovary levels and 30% of fibroblast levels). However, other Cadherins such as CDH2 (at similar levels in cultured ovarian cells compared to ovaries, fibroblasts, and ES cells) and CDH22 (same range as neonatal ovaries, 10-20% of fibroblasts, 50% of ES cells) were expressed by the OCCs. Hence, the phenotypical and molecular indicators of the status of the cells constituting the OCCs are not congruent. In order to initially characterize the features of the OCCs we performed a gene ontology analysis based on the genes up-regulated in OCCs compared to native ovary (Fig.2E). This shows that particularly cell adhesion, ion and neurotransmitter transport as well as signaling pathways are up-regulated in the OCCs.

In summary, the transcriptome data indicate that germ cells are absent from the OCCs. However, the detailed identity of the OCCs remains unclear so far. Due to the limited number of samples (n=2) and the fact that we could analyze only one time point by deep sequencing, we further investigated specific genes by RT-qPCR in different passages to obtain also longitudinal data over the course of the OCC culture.

We have recently shown that the neonatal marmoset monkey ovary contains primitive proliferating germ cells expressing the germ cell and pluripotency markers OCT4A, SALL4, LIN28, and the general germ cell marker VASA (DDX4) [18]. All these markers are only very poorly represented in the transcriptomes of the early passage OCCs or were even absent. We also failed to detect OCT4A, LIN28, or VASA on the protein level in early passage OCC samples by a well-established immunohistochemistry protocol [18] (data not shown). In order to quantify the expression of selected key marker genes in OCCs in relation to ES cells, skin fibroblasts and neonatal ovaries by an independent method and also at higher (>4) passages, we performed RT-qPCR for a number of pluripotency and (premeiotic) germ cell markers including *OCT4A* [25], *NANOG* [25], *SALL4* [26], *LIN28* [27], *VASA* [28; see also Fig. 5], *DAZL* [29], *NOBOX* [30], *DPPA3/STELLA/PGC7* [31, 32], *PRDM1*[33], and *PRDM14* [34, 35]. Fig.3 shows the exemplary RT-qPCR data of one culture from passage 1 to passage 13. These data confirm that the most indicative pluripotency factors *OCT4A* and *NANOG* were not expressed in all OCC samples analyzed (Fig.3). In contrast, *SALL4*, *LIN28* and *VASA* were induced in passages ≥P4 except for SALL4 and LIN28 in P9. In order to further substantiate these findings, we also tested the expression of *PRDM1*, *PRDM14*, *DAZL*, *DPPA3*, *NOBOX*, and *SCP3* in the OCCs from different passages (Fig.4). *DAZL*, *DPPA3*, *NOBOX*, and *SCP3* as specific germ-cell genes were absent or very low at low passages, which is in concordance with the data shown in Fig.3. In later stages, however, all markers were detectable at variable levels. In contrast, *PRDM1* and *PRDM14*, early germ cell specification genes but not specific to germ cells, were robustly expressed in all cell culture samples. These data suggest that our culture supports the survival and probably also the selection of cells that have the potential to (re-)express a set of premeiotic and female germ cell marker genes.

The OCCs generate oocyte-like cells

OCCs generated large, free-floating spherical cells, which we termed oocyte-like cells (OLCs; [17]). OLCs had a morphology resembling immature oocytes. Their diameter ranged from 20 to ~ 40 µm (Fig.5A). We sometimes also observed small structures slightly resembling polar bodies (Fig.5A, right picture). However, we never observed a *Zona pellucida* nor follicle-like structures as described by Dyce, *et al.* [17]. For comparison of the OLCc with real marmoset monkey oocytes, see Fig. 5B. Importantly, OLCs developed even after 20 passages and more than 5 months of culture, but were not observed in passages < 4. We also neither observed OLCs in MEF-only cell cultures nor in ESC cultures which are also based on MEFs. This strongly indicates that the OLCs indeed derive from the OCCs. To initially characterize the OLCs, we tested the expression of key pluripotency and germ cell markers by RT-qPCR. We collected 28 OLCs and randomly allocated the cells to one of two groups (termed OLCs1 and OLCs2), which were then analyzed. *OCT4A*, *NANOG*, and *LIN28* were low in OLCs compared to ES cells and neonatal ovaries (Fig.5C-E). *SALL4* was robustly expressed (Fig.5F) in the range of the controls. In contrast, *PRDM14*, *DPPA3*, *DAZL*, and *VASA*, were much higher in OLCs than in neonatal ovary indicating very robust expression of these germ cell markers in OLCs (Fig.5G-J). *NOBOX* was in a similar range as in the neonatal ovary. Importantly, *DAZL*, *VASA*, and *NOBOX* are specific germ line markers and are all not expressed by ES cells and fibroblasts (Fig.5I-K). As a marker of meiosis, we also tested *SCP3*. This mRNA was also detected in OLCs, although at lower levels compared to the neonatal ovary (Fig.5L). *SCP3* was undetectable in ES cells and fibroblasts. A good indicator of meiosis is chromatin condensation in preparation of the reduction division (Fig.5M, left). However, we did not see a comparable chromatin condensation in OLCs. In contrast, the OLCs showed a homogenous DAPI signal throughout the nucleus (Fig. 5M, right). Although there was no evidence for meiosis, the expression of the markers strongly indicates a germ cell identity of the OLCs. In order to further corroborate the germ line identity of the OLCs we aimed at detecting also VASA protein in the OLCs. First we characterized the VASA

antibody to prevent misleading antibody-based results as we [36] and others [37] recently described. In western blot analysis a very intense and prominent band of the expected size of ~ 85 kDa was detected in testis indicating a high specificity of the VASA antibody (Fig.6A). Lack of a VASA signal in the ovary was due to the fact that the ovary was from an old monkey with an almost exhausted ovarian germ cell reserve. Hence, VASA was below the detection limit in the protein homogenate of the aged ovary. We also tested the VASA antibody in immunohistochemistry on tissue sections from adult marmoset testis and ovary (Fig.6B, C). In both sexes, the antibody very robustly and specifically detected an epitope in germ cells resulting in clear cytoplasmic germ cell labeling. The non-germ cells were not stained or showed only faint background staining. These findings demonstrate the specificity of the antibody also for marmoset VASA protein. We then used this antibody to detect potential OLCs in OCCs. When we fixed OCCs of the 5th passage *in situ*, large cells with strongly condensed chromatin, as indicated by strong DAPI fluorescence in Fig. 6D, were labeled by the VASA antibody (Fig.6E). Whether the signal of the surrounding cells is only background staining or whether it highlights small germ cell progenitor cells cannot be decided at present. The diameter of the large labeled cell was approximately 35 µm like the diameter of the OLCs shown in Fig.5A. We also detached the cell cultures from the culture dish and processed them – like the testis and ovary shown in Fig.6B and C – for immunohistochemistry. We detected large isolated cells that were strongly stained for VASA (Fig.6F, G). The control where VASA was replaced by the corresponding IgG showed only very faint background signals (Fig.6H). These data demonstrate that there are isolated VASA protein-positive oocyte-like cells in the cultures derived from neonatal marmoset monkey ovary.

The relative marker abundance is decreasing within one passage

To obtain initial information on the transcript abundance of the markers during the OCC development over time within one passage, we isolated RNA from duplicate samples from OCCs after 2, 4, 6, and 8 days. *OCT4A, NANOG,* and *LIN28* were very low or absent (Fig.7A-C). In contrast, *SALL4* and *VASA* were robustly detectable in all samples (Fig.7D, E). Both markers exhibited a high abundance at day 2. Later time points (days 4-8) showed a decrease in transcript abundance relative to *GAPDH* probably reflecting a higher proliferation rate of the marker-negative (but *GAPDH* expressing) cells compared to the marker positive cells leading to a dilution effect of the *VASA* and *SALL4*-positive cells.

No production of sex steroids by the OCCs

We were wondering whether the OCCs have the ability to produce female sex steroids like specific cells of the ovary *in vivo*. Therefore, we tested medium samples from low and high culture passages after several days without medium change. Samples were analyzed for estradiol, which is primarily produced by ovarian granulosa cells, and progesterone, which is synthetized by the cells of the *Corpus luteum*. Neither estradiol nor progesterone was detected in medium samples. Moreover, *FSHR* transcripts were undetectable in OCC samples by deep sequencing and *LH/CGR* abundance was lower than in neonatal ovary, fibroblasts and ES cells (data not shown). Hence, the colonies do not consist of functional endocrine cells of the ovary.

Neither teratoma nor ovarian tissue formation in a subcutaneous transplantation assay

Subcutaneous transplantation of cells into immune-deficient mice is a useful approach to assay cells with regard to their differentiation capabilities, e.g. the teratoma formation assay. Moreover, we have recently shown that single-cell suspensions derived from dissociated neonatal monkey testis can reconstitute complex testis tissue after transplantation into immune-deficient mice [24]. In order to test whether the OCCs have the ability to form ovarian

tissue under the "*in vivo*" conditions after transplantation, we injected ~ 10^6 cells per mouse from the 5th passage subcutaneously into 4 female adult NOD-SCID mice. Tissues from the injection site were collected for histological analysis after 15 weeks. Neither ovary-like tissue nor teratoma or any other conspicuous tissue was found (data not shown).

Discussion

A controversial debate on the presence of mitotically active germ line stem cells in the postnatal ovary characterized the last decade of ovarian germ cell research in mammals. Several reports provided data supporting the presence of ovarian germ line stem cells in postnatal mouse ovaries e.g. [10, 13, 38], while other studies failed to identify female germ line stem cells, e.g. [7, 8] thereby supporting the classical view of ovarian biology [1]. In primates including humans, the published data are similarly controversial and still not fully conclusive. Byskov, *et al.* [3] failed to detect oogonia, which may be candidate cells for ovarian stem cells, in the postnatal human ovary older than two years, and even in younger postnatal ovaries the stem cell marker OCT4 was detectable only very rarely. We recently also failed to detect pluripotency and stem cell marker-positive cells in marmoset ovaries at one year of age [18]. On the other hand, White, *et al.* [12] isolated mitotically active cells from human adult ovarian cortex that had the potential to form oocyte-like cells *in vitro* and to form ovarian follicles in a combined allo-xenografting approach. Recently, however, Yuan, *et al.* [9] failed to provide evidence for mitotically active germ line stem cells in rhesus monkeys (*Macaca mulatta*) and mice concluding that adult ovaries do not undergo germ cell renewal. Furthermore, almost ten years ago, Dyce, *et al.* [17] reported that not only ovarian cells have the potential to develop female germ cells, but also fetal porcine skin cells. They formed oocyte-like cells *in vitro* which were extruded from hormone-responsive follicle-like aggregates.

We have established a long-term cell culture system for neonatal marmoset monkey ovarian cells. Seeding of the marmoset monkey ovarian cell suspension onto the MEF cells resulted in the development of cell colonies morphologically resembling colonies which have been observed previously by different other groups for human [39] and mouse [38, 40] ovarian cell cultures. However, while these studies reported the robust expression of pluripotency markers such as OCT4 and NANOG and therefore claimed an ES cell-like character of the cells [40], we

failed to detect these core pluripotency markers in marmoset ovarian cell cultures on the transcript and on the protein level (latter not shown), although our starting material, i.e. neonatal ovary, was positive for these factors. In fact, marmoset monkey primordial germ cells [19] and oogonia [18] robustly express OCT4. This discrepancy between the native premeiotic germ cells and the cultured ovarian cells shows that the marmoset OCCs did not contain remaining (“contaminating”) primordial germ cells or oogonia. All our data point to the fact, that premeiotic germ cells were absent from the early passages of the cultured ovarian cells. This is also in contrast to a publication on pig ovarian stem cells. Bui and colleagues [41] derived putative stem cells from adult pig ovary that gave rise to germ cell-like cells corresponding to primordial germ cells. However, these cells, unlike the cells described in our present study, also robustly expressed pluripotency factors such as *OCT4* and *NANOG*. Moreover, the phenotype of the pig cells in culture was different from the colonies observed in this study and other studies, e.g. [40]. Interestingly, however, despite the clearly different starting conditions, both, our and Bui’s cell culture system resulted in the development of large oocyte-like cells.

In addition to the absence of the pluripotency factors, we did not detect even a single *DAZL*, *MAEL*, *RNF17*, *TEX12*, *TEX101*, or a *TDRD1* transcript in OCCs of the 4th passage by deep sequencing, while all these germ cell transcripts were abundant in the neonatal ovary samples further supporting the absence of germ cells from the marmoset ovarian cell cultures at low passages. Hence, we conclude that marmoset monkey OCCs are neither germ cells nor pluripotent stem cells. The exact identity of the OCCs will be analyzed in future studies. However, although showing morphology resembling an epithelium, the OCCs lacked characteristic proteins of epithelia like E-cadherin and cytokeratins. However, from OCC passage 4 onwards, we observed the development of individual oocyte-like cells strongly expressing the germ cell genes *VASA*, *DAZL*, *NOBOX*, *DPPA3* and *SALL4*. Furthermore, also

the meiotic marker *SCP3* was expressed in OLCs even though at moderate levels. In contrast, *OCT4A*, *NANOG* and *LIN28* were low in OLCs. In addition to the marker expression data on the mRNA level we wanted to confirm VASA also on the protein level. We detected robust VASA protein expression in OLCs. Altogether, this marker profile suggests that the OLCs may correspond to a late premeiotic stage. These characteristics are partly similar to those of the OLCs described by Dyce, *et al.* [17]. However, Dyce and colleagues showed that OLCs developed from cell aggregates that detached from the cell culture surface and formed hormone-responsive follicle-like structures. Then, the OLCs were extruded from the follicles and released into the medium. From ~ 500,000 skin cells approximately 6-70 large cells were extruded. Our findings were in the same range with typically 5-10 large OLCs per well and passage. But we never observed follicle-like structures. Moreover, we did not obtain any evidence for an endocrine regulation of OLC development in the marmoset monkey ovarian cell cultures.

Since we originally intended to culture oogonia, we spun down the cells down primary cells at 200 g. This may be insufficient to quantitatively collect those cells in the pellet, that were cultured in previous studies, where the ovarian germ line stem cells were sedimented at 300g [13] or 1000g [14], respectively. Based on this, we hypothesize that we collected in the present study oogonia (and other larger somatic cells) using a force of 200g but only marginal amounts of the progenitors of the OLCs, which probably are a subpopulation of the OSE [14]. The oogonia apparently did not survive in our culture system. Therefore, we were unable to detect pluripotency markers in our culture and germ cell markers in the early passages. However, we further hypothesize that, despite of the low g force applied in our study some OSE cells with stem cell capacity were still present in the culture. They were hypothetically able to develop into OLCs after more than three passages.

We also tested magnetic activated cell sorting (MACS) to enrich putative stem cells. However, MACS using e.g. TRA-1-81 antibodies was not successful. It must be noted, however, that the neonatal marmoset ovary is extremely tiny and that its availability is very limited. Indeed, the size of the neonatal ovary is only about 2 mm x 1 mm x 1mm. Hence, experimental refinement and cell enrichment approaches are extraordinarily challenging. Therefore, we decided in the present study to culture the unsorted cell population obtained after tissue digestion.

In summary, we have established a long-term neonatal marmoset monkey ovarian cell culture system. However, we failed to detect pluripotent stem and premeiotic germ cell markers in low OCC passages. From passage 4 onwards, however, OLCs developed and still developed at high passages (>20) after more than 5 months of culture. Considering the marker expression data, the view of a germ cell identity of the OLCs appears justified. An essential prerequisite of gamete formation is meiosis [42]. Except for the expression of SCP3, which is an essential protein for meiotic synaptonemal complex formation, we obtained no evidence for meiotic entry of the OLCs except the formation of some structures morphologically resembling polar bodies. We also never observed a *Zona pellucida*. Altogether, OLCs generated in this culture system appear to be germ line cells, but are no functional female gametes nor represent meiotic germ cell stages.

At current it remains to be proven from which progenitor cells the OLCs develop in our culture system. So far, we were not able to identify these cells in the marmoset. However, a candidate tissue for the presence of ovarian stem cells with germ line potential could be the ovarian surface epithelium, which is discussed to be a multipotent tissue possibly harboring a stem or progenitor cell type [43]. In this regard, also the concept of the very small embryonic-like stem cells (VSELs) should be taken into account [16].

Conclusion and outlook

In conclusion, we have established a non-human primate cell culture system that allows the long-term culture and development of OLCs from a non-human primate species. The vast majority of the cultured cells in this study, however, are neither pluripotent stem cells nor germ (line stem) cells, as has been suggested or shown in previous reports [38-40]. Future experiments should aim at resolving this discrepancy and test whether this is a species-specific phenomenon.

Future experiments will also aim at the identification and functional characterization of the stem / progenitor cell population in the marmoset culture system. Furthermore, protocols are needed that support the entry of the OLCs into meiosis potentially giving rise to mature oocytes in the future. Besides functional testing of these cells, their correct epigenetic state needs to be confirmed. In the future, a refined culture system based on the one described here may allow more detailed *in vitro* studies on early phases of primate germ cell development and on the molecular and cellular identity of OLCs and their progenitors in an experimentally accessible NHP system. In the long-term, this may also contribute in the future to the development of novel therapeutic approaches of female infertility.

Acknowledgement

We appreciate the excellent assistance and the support of Nicole Umland, Angelina Berenson and Gesche Spehlbrink. We are also very thankful to Erika Sasaki for providing the ES cells, Katharina Debowski for ES cell culture, and the members of the animal facility for excellent animal husbandry. We thank Ellen Wiese for administrative support. The support of Bentolhoda Fereydouni by the scholarship of the Deutscher Akademischer Austauschdienst (DAAD) is gratefully acknowledged.

Figure Legends

Fig.1: Morphology of ovarian cell colonies (OCCs).

A-C) Morphology of cell colonies in the first passage and in higher passages. **D)** H&E staining of cross sections of colonies. Cells have an epitheloid morphology with rather apical nuclei. **E)** Morphology of a marmoset monkey ES cell colony.

Fig.2: Trancriptome analysis of OCCs.

A) Principal component analysis of the transcriptome analyses of ovarian cell colonies (OCC), neonatal ovaries, which served as starting material for OCC cultures, embryonic stem cells, and fibroblasts. The latter two served as reference samples. OCC transcriptomes differ from the neonatal ovaries´ transcriptomes. However, ovaries, ES cells and OCC are more similar among each other than to fibroblasts (see component 1, relative weight of 38.54%). **B)** Top 50 differentially expressed genes between native neonatal ovary and OCCs. For gene bank and Ensembl identifiers see Supp. Material, Table 1. **C)** Top 50 differentially expressed genes between OCCs and Fibroblasts. **D)** Top 50 differentially expressed genes between OCCs and ES cells. **E)** Gene ontology analysis of OCCS vs. native ovary. Cell adhesion, ion and neurotransmitter transporters as well as signaling pathways are predominantly upregulated in OCCs compared to the native ovary.

Fig.3: RT-qPCR analysis of OCCs in different passages.

Real-time quantitative RT-PCR analysis of selected key pluripotency and germ cell markers in OCCs. *OCT4*, *NANOG*, *SALL4*, and *LIN28* are all robustly expressed in pluripotent stem cells as well as in primitive germ cells. In contrast*OCT4* and *NANOG* are absent from OCCs. *SALL4* and *LIN28* were absent or very low at low passages and increased in expression at

higher passages. *VASA* is a general germ cell marker and was detected in later OCC passages and in the neonatal ovary.

Fig.4: Expression of additional markers in OCCs.

PRDM1 and *PRDM14* are necessary for germ cell specification and are expressed in all OCC passages in the range of the controls. *DAZL* and *DPPA3* are also necessary for germ cell specification and were detectable only is few samples. *NOBOX* is a female-specific germ cell marker and was detectable at relatively low levels in most samples. *SCP3* is a meiosis marker and was detectable only is some samples at variable levels. In general, the germ cell-specific markers were very low or absent during the first three passages..

Fig.5: Oocyte-like cells derived from OCCs highly express germ cell markers.

A) Oocyte-like cells that spontaneously developed even in high cell culture passages. The cells were freely floating in the cell culture medium and had a diameter of approximately 40µm. The passage number is indicated in the upper right corner of each picture. Sometimes small spherical structures attached to the OCLs could be seen slightly resembling polar bodies (arrow). B) Left: A group of natural marmoset monkey oocytes. Some oocytes are still associated with granulosa cells. Right: higher magnification of a marmoset monkey oocyte with a robust *Zona pellucida*. **C-L)** Oocyte-like cells express pluripotency and germ cell markers. Relative mRNA levels of selected pluripotency and germ cell markers in oocyte-like cells as revealed by real-time quantitative RT-PCR. For *OCT4A*, *SALL4*, and *LIN28*, ES cells were used as positive control. Neonatal ovary was used as positive control for the germ cell markers. Fibroblasts served as biological negative control. **M)** DAPI staining of a natural oocyte (left) and of a OLC. While the oocyte shows strongly compacted chromatin, the DAPI

staining of the OLC is homogenous indicating a different chromatin state in OLCs and natural oocytes.

Fig.6: Characterization of the VASA antibody and detection of VASA-positive cells in OCCs.

A) Western blot analysis using marmoset monkey protein samples demonstrates the specificity of the VASA antibody. * indicates that the ovary was from an aged animal. The ovarian germ cell pool was therefore most likely strongly reduced or even exhausted leading to undetectable VASA protein concentrations in the sample. **B and C)** Immunohistochemical application of the VASA antibody to marmoset gonads showed germ cell-specific labeling in the adult testis and the adult ovary. **D)** DAPI staining of a part of an OCC. Note the intensely stained nucleus in the central part. **E)** Same area shown in D. The large cell containing the intensely stained nucleus strongly stains for VASA. The signal is blurry due to the fact that the picture was taken through the bottom of a normal cell culture dish. **F, G)** Examples of isolated VASA-positive cells in sections of paraffin-embedded OCCs.

Fig.7: Pluripotency and germ cell marker mRNA abundance during the development of OCCs within one passage as revealed by real-time quantitative PCR. Positive controls for *OCT4A*, *SALL4*, and *LIN28* were ES cells. Neonatal ovary was used as positive control for the germ cell marker *VASA*. *NANOG* was very low and *OCT4A* and *LIN28* were absent at all time points analyzed. Relative abundance of *SALL4* and *VASA* generally decreased with time during the culture period.

Fig. S1: Number of reads per biological sample in transcriptome analysis. Between 13 Mio and 19 Mio reads were analyzed.

Fig. S2: Number of transcripts detected per biological sample in transcriptome analysis. In all samples around 40,000 different transcripts were detected.

Fig.S3: Ki-67 staining of P1 (left) and P9 (middle) OCCs. ES cells were used as positive control (right). The staining shows that the OCCs show robust proliferation at P1 and reduced Ki-67 labeling at P9. Almost all ES cells were Ki-67-positive.

Table 1: Primer sequences, sizes of amplicons and concentration of respective primers.

Primer	Primer sequence	PCR product size (bp)	Concentration [nM]
Cj_*GAPDH*_Fw	5'-TGCTGGCGCTGAGTATGTG-3'	64	300
Cj_*GAPDH*_Re	5'-AGCCCCAGCCTTCTCCAT-3'		50
Cj_*LIN28*_Fw	5'-GACGTCTTTGTGCACCAGAGTAA-3'	67	300
Cj_*LIN28*_Re	5'-CGGCCTCACCTTCCTTCAA-3'		50
Cj_*SALL4*_Fw	5'-AAGGCAACTTGAAGGTTCACTACA-3'	77	900
Cj_*SALL4*_Re	5'-GATGGCCAGCTTCCTTCCA-3'		50
Cj_*VASA*_Fw	5'-TGGACATGATGCACCACCAGCA-3'	210	50
Cj_*VASA*_Re	5'-TGGGCCAAAATTGGCAGGAGAAA-3'		900
Cj_*OCT4A*_Fw	5'-GGAACAAAACACGGAGGAGTC-3'	234	300
Cj_*OCT4A*_Re	5'-CAGGGTGATCCTCTTCTGCTTC-3'		50
Cj_ *PRDM1*_Fw	5'- ATGAAGTTGCCTCCCAGCAA-3'	147	50
Cj_*PRDM1*_Re	5'- TTCCTACAGGCACCCTGACT-3'		50
Cj_*PRDM14*_Fw	5´- CGGGGAGAAGCCCTTCAAAT -3´	91	50
Cj_*PRDM14*_Re	5´- CTCCTTGTGTGAACGTCGGA -3´		50
Cj_ *DAZL* _Fw	5'- GAAGAAGTCGGGCAGTGCTT -3'	70	50
Cj_ *DAZL* _Re	5'- AACGAGCAACTTCCCATGAA-3'		50
Cj_ *DPPA3*_Fw	5'- GCGGATGGGATCCTTCTGAG -3'	129	50
Cj_ *DPPA3*_Re	5'- GAGTAGCTTTCTCGGTCTGCT -3'		50
Cj_*NOBOX*_Fw	5'- GAAGACCACTATCCTGACAGTG-3'	320	50
Cj_*NOBOX*_Re	5'- TCAGAAGTCAGCAGCATGGGG-3'		50
*Cj _SCP3*_Fw	5'- TGGAAAACACAACAAGATCA-3'	60	50
Cj _SCP3-Rw	5'- GCTATCTCTTGCTGCTGAGT -3'		50

Supp. Table 1:

Ovary vs. OCCs top 50 up-regulated genes corresponding to data base identifier

LOC100397701	MOV10L1 Mov10l1, Moloney leukemia virus 10-like 1, homolog (mouse) [Callithrix jacchus (white-tufted-ear marmoset)
LOC100398487	(homeobox protein NOBOX-like [Callithrix jacchus (white-tufted-ear marmoset)])
ENSCJAT00000031636	zona pellucida glycoprotein 3 (ZP3)
LOC100411838	factor in the germline alpha-like [Callithrix jacchus (white-tufted-ear marmoset)]
LOC100414988	TKTL1 transketolase-like 1 [Callithrix jacchus (white-tufted-ear marmoset)]
LOC100399713	TYR tyrosinase (oculocutaneous albinism IA) [Callithrix jacchus (white-tufted-ear marmoset)]
LOC100394179	DKK4 dickkopf homolog 4 (Xenopus laevis) [Callithrix jacchus (white-tufted-ear marmoset)]
ENSCJAT00000037797	Novel gene
LOC100392679	SOHLH2 spermatogenesis and oogenesis specific basic helix-loop-helix 2 [Callithrix jacchus (white-tufted-ear marmoset)]
LOC100388137	C12H16orf73 chromosome 12 open reading frame, human C16orf73 [Callithrix jacchus (white-tufted-ear marmoset)]
ENSCJAT00000006890	Deleted in azoospermia-like (DAZL)
LOC100412246	RAB41, member RAS oncogene family [Callithrix jacchus (white-tufted-ear marmoset)]
LOC100408598	HORMAD1 HORMA domain containing 1 [Callithrix jacchus (white-tufted-ear marmoset)]
LOC100399315	uncharacterized LOC100399315 [Callithrix jacchus (white-tufted-ear marmoset)]
LOC100415170	F-box/WD repeat-containing protein 12-like [Callithrix jacchus (white-tufted-ear marmoset)]
LOC100415174	NYAP1 neuronal tyrosine-phosphorylated phosphoinositide-3-kinase adaptor 1 [Callithrix jacchus (white-tufted-ear marmoset)]
ENSCJAT00000037799	Novel gene
LOC100402230	PLAC1 placenta-specific 1 [Callithrix jacchus (white-tufted-ear marmoset)]
LOC100400731	glutathione S-transferase A3-like [Callithrix jacchus (white-tufted-ear marmoset)]
ENSCJAT00000053281	Novel gene
LOC100400032	PNMA5 paraneoplastic Ma antigen family member 5 [Callithrix jacchus (white-tufted-ear marmoset)]
LOC100391376	beta-galactosidase-1-like protein 3-like [Callithrix jacchus (white-tufted-ear marmoset)

1. Zuckerman S. The number of oocytes in the mature ovary. Recent Prog Horm Res. 1951;6:63-108.
2. Byskov AG, Faddy MJ, Lemmen JG, et al. Eggs forever? Differentiation. 2005;73:438-446.
3. Byskov AG, Hoyer PE, Yding Andersen C, et al. No evidence for the presence of oogonia in the human ovary after their final clearance during the first two years of life. Hum Reprod. 2011;26:2129-2139.
4. Liu Y, Wu C, Lyu Q, et al. Germline stem cells and neo-oogenesis in the adult human ovary. Dev Biol. 2007;306:112-120.
5. Bendsen E, Byskov AG, Andersen CY, et al. Number of germ cells and somatic cells in human fetal ovaries during the first weeks after sex differentiation. Hum Reprod. 2006;21:30-35.
6. Stoop H, Honecker F, Cools M, et al. Differentiation and development of human female germ cells during prenatal gonadogenesis: an immunohistochemical study. Hum Reprod. 2005;20:1466-1476.
7. Zhang H, Zheng W, Shen Y, et al. Experimental evidence showing that no mitotically active female germline progenitors exist in postnatal mouse ovaries. Proc Natl Acad Sci U S A. 2012;109:12580-12585.
8. Lei L, Spradling AC. Female mice lack adult germ-line stem cells but sustain oogenesis using stable primordial follicles. Proc Natl Acad Sci U S A. 2013;110:8585-8590.
9. Yuan J, Zhang D, Wang L, et al. No evidence for neo-oogenesis may link to ovarian senescence in adult monkey. Stem Cells. 2013;31:2538-2550.
10. Johnson J, Canning J, Kaneko T, et al. Germline stem cells and follicular renewal in the postnatal mammalian ovary. Nature. 2004;428:145-150.
11. Johnson J, Bagley J, Skaznik-Wikiel M, et al. Oocyte generation in adult mammalian ovaries by putative germ cells in bone marrow and peripheral blood. Cell. 2005;122:303-315.
12. White YA, Woods DC, Takai Y, et al. Oocyte formation by mitotically active germ cells purified from ovaries of reproductive-age women. Nat Med. 2012;18:413-421.
13. Zou K, Yuan Z, Yang Z, et al. Production of offspring from a germline stem cell line derived from neonatal ovaries. Nat Cell Biol. 2009;11:631-636.
14. Bukovsky A, Svetlikova M, Caudle MR. Oogenesis in cultures derived from adult human ovaries. Reprod Biol Endocrinol. 2005;3:17.
15. Virant-Klun I, Zech N, Rozman P, et al. Putative stem cells with an embryonic character isolated from the ovarian surface epithelium of women with no naturally present follicles and oocytes. Differentiation. 2008;76:843-856.
16. Parte S, Bhartiya D, Telang J, et al. Detection, characterization, and spontaneous differentiation in vitro of very small embryonic-like putative stem cells in adult mammalian ovary. Stem Cells Dev. 2011;20:1451-1464.
17. Dyce PW, Wen L, Li J. In vitro germline potential of stem cells derived from fetal porcine skin. Nat Cell Biol. 2006;8:384-390.
18. Fereydouni B, Drummer C, Aeckerle N, et al. The neonatal marmoset monkey ovary is very primitive exhibiting many oogonia. Reproduction. 2014;148:237-247.
19. Aeckerle N, Drummer C, Debowski K, et al. Primordial germ cell development in the marmoset monkey as revealed by pluripotency factor expression: suggestion of a novel model of embryonic germ cell translocation. Mol Hum Reprod. 2015;21:66-80.
20. Mueller T, Fleischmann G, Eildermann K, et al. A novel embryonic stem cell line derived from the common marmoset monkey (*Callithrix jacchus*) exhibiting germ cell-like characteristics. Hum Reprod. 2009;24:1359-1372.
21. Debowski K, Warthemann R, Lentes J, et al. Non-viral generation of marmoset monkey iPS cells by a six-factor-in-one-vector approach. PLoS One. 2015;10:e0118424.
22. Heistermann M, Tari S, Hodges JK. Measurement of faecal steroids for monitoring ovarian function in New World primates, Callitrichidae. J Reprod Fertil. 1993;99:243-251.

23. Heistermann M, Finke M, Hodges JK. Assessment of female reproductive status in captive-housed Hanuman langurs (Presbytis entellus) by measurement of urinary and fecal steroid excretion patterns. Am J Primatol. 1995;37:275-284.
24. Aeckerle N, Dressel R, Behr R. Grafting of Neonatal Marmoset Monkey Testicular Single-Cell Suspensions into Immunodeficient Mice Leads to ex situ Testicular Cord Neomorphogenesis. Cells Tissues Organs. 2013;198:209-220.
25. Perrett RM, Turnpenny L, Eckert JJ, et al. The early human germ cell lineage does not express SOX2 during in vivo development or upon in vitro culture. Biol Reprod. 2008;78:852-858.
26. Eildermann K, Aeckerle N, Debowski K, et al. Developmental expression of the pluripotency factor sal-like protein 4 in the monkey, human and mouse testis: restriction to premeiotic germ cells. Cells Tissues Organs. 2012;196:206-220.
27. Aeckerle N, Eildermann K, Drummer C, et al. The pluripotency factor LIN28 in monkey and human testes: a marker for spermatogonial stem cells? Mol Hum Reprod. 2012;18:477-488.
28. Castrillon DH, Quade BJ, Wang TY, et al. The human VASA gene is specifically expressed in the germ cell lineage. Proc Natl Acad Sci U S A. 2000;97:9585-9590.
29. Lin Y, Gill ME, Koubova J, et al. Germ cell-intrinsic and -extrinsic factors govern meiotic initiation in mouse embryos. Science. 2008;322:1685-1687.
30. Rajkovic A, Pangas SA, Ballow D, et al. NOBOX deficiency disrupts early folliculogenesis and oocyte-specific gene expression. Science. 2004;305:1157-1159.
31. Liu YJ, Nakamura T, Nakano T. Essential role of DPPA3 for chromatin condensation in mouse oocytogenesis. Biol Reprod. 2012;86:40.
32. Sato M, Kimura T, Kurokawa K, et al. Identification of PGC7, a new gene expressed specifically in preimplantation embryos and germ cells. Mech Dev. 2002;113:91-94.
33. Ohinata Y, Payer B, O'Carroll D, et al. Blimp1 is a critical determinant of the germ cell lineage in mice. Nature. 2005;436:207-213.
34. Yamaji M, Seki Y, Kurimoto K, et al. Critical function of Prdm14 for the establishment of the germ cell lineage in mice. Nat Genet. 2008;40:1016-1022.
35. Sugawa F, Arauzo-Bravo MJ, Yoon J, et al. Human primordial germ cell commitment in vitro associates with a unique PRDM14 expression profile. EMBO J. 2015;34:1009-1024.
36. Warthemann R, Eildermann K, Debowski K, et al. False-positive antibody signals for the pluripotency factor OCT4A (POU5F1) in testis-derived cells may lead to erroneous data and misinterpretations. Mol Hum Reprod. 2012;18:605-612.
37. Ivell R, Teerds K, Hoffman GE. Proper application of antibodies for immunohistochemical detection: antibody crimes and how to prevent them. Endocrinology. 2014;155:676-687.
38. Pacchiarotti J, Maki C, Ramos T, et al. Differentiation potential of germ line stem cells derived from the postnatal mouse ovary. Differentiation. 2010;79:159-170.
39. Stimpfel M, Skutella T, Cvjeticanin B, et al. Isolation, characterization and differentiation of cells expressing pluripotent/multipotent markers from adult human ovaries. Cell Tissue Res. 2013;354:593-607.
40. Gong SP, Lee ST, Lee EJ, et al. Embryonic stem cell-like cells established by culture of adult ovarian cells in mice. Fertil Steril. 2010;93:2594-2601, 2601 e2591-2599.
41. Bui HT, Van Thuan N, Kwon DN, et al. Identification and characterization of putative stem cells in the adult pig ovary. Development. 2014;141:2235-2244.
42. Handel MA, Eppig JJ, Schimenti JC. Applying "gold standards" to in-vitro-derived germ cells. Cell. 2014;157:1257-1261.
43. Bukovsky A, Caudle MR, Virant-Klun I, et al. Immune physiology and oogenesis in fetal and adult humans, ovarian infertility, and totipotency of adult ovarian stem cells. Birth Defects Res C Embryo Today. 2009;87:64-89.
