# Supplementary material for: Long-Term Oocyte-Like Cell Development in Cultures Derived from Neonatal Marmoset Monkey Ovary
Source: Stem Cells Int. 2015 Nov 9;2016:2480298. doi: 10.1155/2016/2480298 (PMC4655298; doi:10.1155/2016/2480298)
Supplement: Supplementary file 1 — Supplementary Figure 1. The number of reads per sample analyzed in this study by transcriptome analysis. ES cells (samples 1-4), fibroblasts (samples 5-10), ovarian cell culture samples (samples 11 and 12), and native marmoset monkey neonatal ovaries (samples 13 and 14) were analyzed. The numbers of reads per sample were between 13 and 19 × 106. Supplementary Figure 2. The number of detected transcripts per sample analyzed in this study by transcriptome analysis. Around 40.000 transcripts were detected in all samples [ES cells (samples 1-4), fibroblasts (samples 5-10), ovarian cell culture samples (samples 11 and 12), and native neonatal ovaries (samples 13 and 14)]. Supplementary Figure 3. The cultured ovarian cells were highly proliferative at low passages (P1, left panel) as revealed by Ki-67 staining. At higher passages (P9), OCCs showed a reduced number of Ki-67-positive cells. Highly proliferative ES cells were used as positive control. The primary antibody (#9027S from Cell signaling Technology) was used in a 1:300 dilution. The scale bar represents 50 μm. Supplementary Table 1. Ovary vs. OCCs top 50 up-regulated genes corresponding to data base identifier. [file 2480298.f1.zip › 2480298.f1/mat.102894.v2.docx]

Fereydouni et al.

Short description of the Supplementary Material:

Supp. Material Fig.1:

The number of reads per sample analyzed in this study by transcriptome analysis.

ES cells (samples 1-4), fibroblasts (samples 5-10), ovarian cell culture samples (samples 11 and 12), and native marmoset monkey neonatal ovaries (samples 13 and 14) were analyzed. The numbers of reads per sample were between 13 and 19 x 10^6^.

Supp. Material Fig.2:

The number of detected transcripts per sample analyzed in this study by transcriptome analysis.

Around 40.000 transcripts were detected in all samples [ES cells (samples 1-4), fibroblasts (samples 5-10), ovarian cell culture samples (samples 11 and 12), and native neonatal ovaries (samples 13 and 14)].

Supp. Material Fig.3:

The cultured ovarian cells were highly proliferative at low passages (P1, left panel) as revealed by Ki-67 staining. At higher passages (P9), OCCs showed a reduced number of Ki-67-positive cells. Highly proliferative ES cells were used as positive control. The primary antibody (#9027S from Cell signaling Technology) was used in a 1:300 dilution. The scale bar represents 50 μm.

**Supp. Table 1:** 666

**Ovary vs. OCCs top 50 up-regulated genes corresponding to data base identifier**
