# Supplementary material for: Long‐Term Oocyte‐Like Cell Development in Cultures Derived from Neonatal Marmoset Monkey Ovary
Source: Stem Cells Int. 2015 Nov 9;2016:2480298. doi: 10.1155/2016/2480298 (PMC4655298; doi:10.1155/2016/2480298)
Supplement: Supplementary file 6 [file SCI-2016-2480298-s001.zip › 2480298.f1/Supplementary_Table_1.pdf]

Supp. Table 1:

Ovary vs. OCCs top 50 up-regulated genes corresponding to data base identifier

LOC100397701	MOV10L1 Mov10l1, Moloney leukemia virus 10-like 1, homolog (mouse) [Callithrix jacchus (white-tufted-ear marmoset)
LOC100398487	(homeobox protein NOBOX-like [Callithrix jacchus (white-tufted-ear marmoset)])
ENSCJAT00000031636	zona pellucida glycoprotein 3 (ZP3)
LOC100411838	factor in the germline alpha-like [Callithrix jacchus (white-tufted-ear marmoset)]
LOC100414988	TKTL1 transketolase-like 1 [Callithrix jacchus (white-tufted-ear marmoset)]
LOC100399713	TYR tyrosinase (oculocutaneous albinism IA) [Callithrix jacchus (white-tufted-ear marmoset)]
LOC100394179	DKK4 dickkopf homolog 4 (Xenopus laevis) [Callithrix jacchus (white-tufted-ear marmoset)]
ENSCJAT00000037797	Novel gene
LOC100392679	SOHLH2 spermatogenesis and oogenesis specific basic helix-loop-helix 2 [Callithrix jacchus (white-tufted-ear marmoset)]
LOC100388137	C12H16orf73 chromosome 12 open reading frame, human C16orf73 [Callithrix jacchus (white-tufted-ear marmoset)]
ENSCJAT00000006890	Deleted in azoospermia-like (DAZL)
LOC100412246	RAB41, member RAS oncogene family [Callithrix jacchus (white-tufted-ear marmoset)]
LOC100408598	HORMAD1 HORMA domain containing 1 [Callithrix jacchus (white-tufted-ear marmoset)]
LOC100399315	uncharacterized LOC100399315 [Callithrix jacchus (white-tufted-ear marmoset)]
LOC100415170	F-box/WD repeat-containing protein 12-like [Callithrix jacchus (white-tufted-ear marmoset)]
LOC100415174	NYAP1 neuronal tyrosine-phosphorylated phosphoinositide-3-kinase adaptor 1 [Callithrix jacchus (white-tufted-ear marmoset)]
ENSCJAT00000037799	Novel gene
LOC100402230	PLAC1 placenta-specific 1 [Callithrix jacchus (white-tufted-ear marmoset)]
LOC100400731	glutathione S-transferase A3-like [Callithrix jacchus (white-tufted-ear marmoset)]
ENSCJAT00000053281	Novel gene
LOC100400032	PNMA5 paraneoplastic Ma antigen family member 5 [Callithrix jacchus (white-tufted-ear marmoset)]
LOC100391376	beta-galactosidase-1-like protein 3-like [Callithrix jacchus (white-tufted-ear marmoset)
